# Serological assessment of neutrophil elastase activity on elastin during lung ECM remodeling

**DOI:** 10.1186/s12890-015-0048-5

**Published:** 2015-05-03

**Authors:** Jacob H Kristensen, Morten A Karsdal, Jannie MB Sand, Nicholas Willumsen, Claudia Diefenbach, Birte Svensson, Per Hägglund, Diana J Oersnes-Leeming

**Affiliations:** Nordic Bioscience A/S, Herlev, Denmark; Department of Systems Biology, The Technical University of Denmark, Kgs. Lyngby, Denmark; Boehringer-Ingelheim Pharma GmbH, Biberach, Germany

**Keywords:** Biomarker, Neutrophil elastase, IPF, ECM, Lung cancer, Elastin

## Abstract

**Background:**

During the pathological destruction of lung tissue, neutrophil elastase (NE) degrades elastin, one of the major constituents of lung parenchyma. However there are no non-invasive methods to quantify NE degradation of elastin. We selected specific elastin fragments generated by NE for antibody generation and developed an ELISA assay (EL-NE) for the quantification of NE-degraded elastin.

**Methods:**

Monoclonal antibodies were developed against 10 NE-specific cleavage sites on elastin. One EL-NE assay was tested for analyte stability, linearity and intra- and inter-assay variation. The NE specificity was demonstrated using elastin cleaved *in vitro* with matrix metalloproteinases (MMPs), cathepsin G (CatG), NE and intact elastin. Clinical relevance was assessed by measuring levels of NE-generated elastin fragments in serum of patients diagnosed with idiopathic pulmonary fibrosis (IPF, n = 10) or lung cancer (n = 40).

**Results:**

Analyte recovery of EL-NE for human serum was between 85% and 104%, the analyte was stable for four freeze/thaw cycles and after 24 h storage at 4°C. EL-NE was specific for NE-degraded elastin. Levels of NE-generated elastin fragments for elastin incubated in the presence of NE were 900% to 4700% higher than those seen with CatG or MMP incubation or in intact elastin. Serum levels of NE-generated elastin fragments were significantly increased in patients with IPF (137%, p = 0.002) and in patients with lung cancer (510%, p < 0.001) compared with age- and sex-matched controls.

**Conclusions:**

The EL-NE assay was specific for NE-degraded elastin. The EL-NE assay was able to specifically quantify NE-degraded elastin in serum. Serum levels of NE-degraded elastin might be used to detect excessive lung tissue degradation in lung cancer and IPF.

**Electronic supplementary material:**

The online version of this article (doi:10.1186/s12890-015-0048-5) contains supplementary material, which is available to authorized users.

## Background

The common denominator of several lung diseases is massive lung extracellular matrix (ECM) remodeling [1]. Lung diseases with increased lung tissue turnover include idiopathic pulmonary fibrosis (IPF) and lung cancer [2,3]. Protease-mediated degradation of the lung ECM is a well documented and a significant part of lung tissue degradation [1,4]. Many of the responsible proteases, neutrophils in particular, are associated with lung diseases such as IPF and lung cancer [5,6].

One of the signature proteins of the lungs is elastin. Elastin provides resilience and elasticity to the lungs [7,8]. Neutrophil elastases (NEs) are produced by neutrophils [9] and are able to degrade the otherwise stable elastin fibers in the lung [10]. NE expression and activity levels have for some time been coupled with chronic obstructive pulmonary disease (COPD). Neutrophil levels identified in the sputum of patients diagnosed with COPD have been shown to be elevated compared with controls [9]. In alignment, an increase in neutrophil cell count and cell percentage was observed in bronchoalveolar fluid (BALF) from patients with IPF compared with controls [5]. Furthermore NE may play a destructive role in lung cancer especially non-small-cell lung cancer subtypes such as squamous cell carcinoma and adenocarcinoma [11].

The aim of this study was to identify and quantify NE-generated elastin degradation, and assess the relationship between levels of elastin fragments and pulmonary disorders such as lung cancer and IPF that involve excessive lung tissue remodeling. We selected unique fragments of elastin degraded specifically by NE, developed a novel ELISA assay detecting exclusively NE-degraded elastin and investigated NE activity towards elastin in lung cancer and IPF.

## Methods

### Selection of peptides for immunizations

Decapeptides at the N- or C-terminus of known NE cleavage sites on elastin [10] were considered candidate immunogens. The decapeptides were selected for their distance to other known elastase cleavage sites in the primary structure of elastin [10,12]. Cleavage sites furthest away from other cleavage sites were preferred and the final number of candidates was reduced to 10. Selected decapeptide sequences were screened for theoretical cleavage sites of trypsin and chymotrypsin as well as to the cleavage sites from in-house human elastin degradome database obtained from previous study [13]. Selected decapeptides were blasted for homology to decapeptide sequences from other proteins using the NPS@: network protein sequence analysis with the UniProt/Swiss-Prot database [14]. A total of 60 mice were immunized, with groups of six mice each being immunized with a single immunogen.

### Immunization procedure

The immunization [13] and fusion procedures [15] have been described elsewhere. To create the EL-NE assay immunogen, the immunogen (CGG-GGPGFGPGVV, Chinese Peptide Company, Beijing, China) was coupled to the Keyhole limpet hemocyanin (KLH) carrier protein using Succinimidyl-4-(N-maleimidomethyl)cyclohexane-1-carboxylate (SMCC, Thermo Scientific, Waltham, MA, USA) as the linker. The adjuvant used was Freund’s incomplete adjuvant (Sigma-Aldrich, St. Louis, MO, USA)

### Characterization and selection of antibodies

Native reactivity and peptide binding of the generated monoclonal antibodies were validated by displacement of human serum in a preliminary indirect ELISA using biotinylated peptides (Biotin-KK-GGPGFGPGVV for EL-NE) on a streptavidin-coated microtitre plate (Roche, Basel, Switzerland) and the supernatant from the monoclonal hybridoma. The clones were characterized by testing the reactivity of their supernatant to the free peptide (GGPGFGPGVV for EL-NE), the elongated peptide (GGPGFGPGVVG for EL-NE) and a nonsense peptide (VGAGVPGLGV for EL-NE). The selected clones were purified using Protein G columns according to manufacturer’s instructions (GE Healthcare Life Science, Little Chalfont, Buckinghamshire, UK). All peptides were produced by the Chinese Peptide Company (Beijing, China). The final selection of the monoclonal antibody for assay development was based on high reactivity towards its free peptide and towards serum samples from patients diagnosed with IPF (ProteoGenex, Culver City, CA, USA) combined with low reactivity towards their elongated peptide and intact elastin. Peptide sequences for applied peptides were confirmed with mass spectrometry by the peptide company (Chinese Peptide Company, Beijing, China) after synthesis.

### EL-NE protocol

The selected monoclonal antibody was inserted into competitive ELISA systems and labeled with horseradish peroxidase (HRP) using the Lightning link HRP labeling kit according to the instructions of the manufacturer (cat. no.701-003 Innova Bioscience, Babraham, Cambridge, UK). A 96-well streptavidin plate was coated with 5 ng/mL screening peptide (Biotin-KK-GGPGFGPGVV for EL-NE) dissolved in coater buffer (10 mM PBS, 1% BSA, 0.1% Tween-20, pH 7.4, 8 g/L NaCl) and incubated for 30 minutes at 20°C. 20 μL of free peptide calibrator or sample were added in duplicate to appropriate wells, followed by 100 μL of conjugated monoclonal antibody in assay buffer (25 mM PBS, 1% BSA, 0.1% Tween-20, pH 7.4, 2 g/L NaCl) and incubated for 3 hours at 4°C. After each incubation step the plate was washed five times in washing buffer (20 mM Tris, 50 mM NaCl, pH 7.2). Finally, 100 μL tetramethylbenzinidine (TMB, Kem-En-Tec Nordic, Taastrup, Denmark) was added and the plate was incubated for 15 minutes at 20°C in the dark. All the above incubation steps included shaking at 300 rpm. The TMB reaction was stopped by adding 100 μL of stopping solution (1% H_2_SO_4_) and optical density was measured at 450 nm with 650 nm as the reference.

### Technical validation of EL-NE

From 2-fold dilutions of human samples of serum and plasma citrate and heparin, linearity was calculated as a percentage of recovery of the undiluted neoepitope. The lower limit of detection was determined from 21 zero samples (assay buffer) and calculated as the mean + 3X standard deviation*.* The lower limit of quantification was determined as the highest level of NE-generated elastin fragments with coefficient of variation (CV) below 30% reproduced in serum samples. The inter- and intra-assay variation was determined by 10 independent runs of 8 samples that covered the detection range of the EL-NE. Besides five human serum samples, the 8 samples included one bovine serum sample, one sample with the free peptide in human serum and one sample with the free peptide in buffer. The freeze-thaw recovery of human serum and citrate and heparin plasma was determined by measuring the NE-degraded levels of elastin in three samples of each, which were exposed to four freeze-thaw cycles and compared to NE-generated levels of elastin prior to the first cycle. Analyte stability was determined by the levels of NE-degraded elastin in three samples each of human serum and plasma citrate and heparin after either 4°C or 20°C storage for 24 hours and compared with the levels at zero hours.

### EL-NE specificity

The reactivity of the EL-NE antibody towards the free peptide (GGPGFGPGVV) was compared with its reactivity to the elongated peptide (GGPGFGPGVVG), a nonsense peptide (VGAGVPGLGV) as well as to the free peptide where a nonsense peptide was applied as screening peptide (VGAGVPGLGV-KK-Biotin). The added peptide doses were 119 nM, 59 nM, 30 nM, 15 nM, 7 nM, 4 nM, 2nM and 0 nM.

Levels of NE-degraded elastin were determined in the presence of elastin *in vitro* cleaved with: matrix metalloproteinase (MMP)-2, MMP-7, MMP-9, MMP-12 or NE, NE in NE buffer as well as intact elastin dissolved in NE buffer (all incubated for 48 hours at 37°C). Elastin was incubated once with each enzyme. Enzyme:protein ratios were 1:100 (MMPs) or 1:200 (NE) (weight/weight). For cross-reactivity towards CatG cleavage, enzyme:protein ratios were 1:50 (NE) and 1:15 (CatG) (weight/weight). Incubation times for the cleavages, intact elastin, NE and CatG were 24 hours at 37°C. Activity tests were performed on proteases prior to cleavage. All material was diluted 100x in assay buffer before measurement. Insoluble elastin was purchased from Sigma-Aldrich (cat. no. E6777, St. Louis, MO, USA); MMP-2 and MMP-9 from Calbiochem (cat. no. 444213 and 444231, Whitehouse Station, NJ, USA), MMP-7 and MMP-12 from R&D Systems (cat. no. 907-MP-010 and 917-MP-010, Minneapolis, MN, USA), cathepsin G from Elastin Product Company (cat. No. SG623, Owensville, MO, USA) and NE from Abcam (cat. no. ab80475, Cambridge, UK).

### Clinical validation of EL-NE

Levels of NE-degraded elastin were determined in serum from patients diagnosed with IPF (n = 10, mean age 74 years, 20% female) and compared with healthy age- and sex-matched controls (n = 9, mean age 72 years, 22% female). NE-generated elastin levels were also measured in serum from patients diagnosed with lung cancer (n = 40, of which n = 16 had squamous cell carcinoma, n = 16 had adenocarcinoma, n = 8 had small cell lung cancer; mean age 59 years, 25% female) and compared with healthy age- and sex-matched controls (n = 12, mean age 60 years, 25% female). All controls were derived from a previously described study [16]. Patient samples were obtained from the commercial vendor Proteogenex (Culver City, CA). After signed consent from patients and approval by the appropriate Institutional Review Board or Independent Ethical Committee, serum had been collected from patients with IPF or lung cancer. According to Danish law, it is not required to obtain ethical approval when measuring biochemical markers in previously collected samples; hence, there was no additional ethical approval for this study. Samples were all collected, processed, and stored in a similar fashion until analyzed. Patient samples were collected prior to surgery. Additional patient demographics and clinical information is presented in table S1 (see Additional file 1: Appendix 1).

### Statistical analyses

The geometric means (95% CI) of serum levels of NE-generated elastin fragments in diagnosed patients were compared with their respective controls using the two-sided non-parametric Mann Whitney test. All statistical analyses were performed in MedCalc from MedCalc Software (Ostend, Belgium). Results were considered statistically significant if p < 0.05.

Detailed materials and methods for the EL-NE-B competitive ELISA (VGAGVPGLGV) can be seen in Additional file 2: Appendix 1.

## Results

### Selection, reactivity, specificity and technical validation of the EL-NE assay

The EL-NE sequence (GGPGFGPGVV) was unique in the human proteome with homology to human elastin [UniProt:P15502] only. Ten decapeptides were selected for immunization after the homology search, from which a total of 60 antibodies were developed. The EL-NE antibody was selected due to its high reactivity towards free peptide, elastin cleaved with NE and serum from patients diagnosed with IPF and lung cancer, combined with a low reactivity towards the elongated peptide, intact elastin as well as elastin cleaved with MMPs. Results from a different decapeptide immunogen candidate, EL-NE-B, are shown in Additional file 2: Appendix 2, Figure S1 and Figure S2.

**Figure 1:**
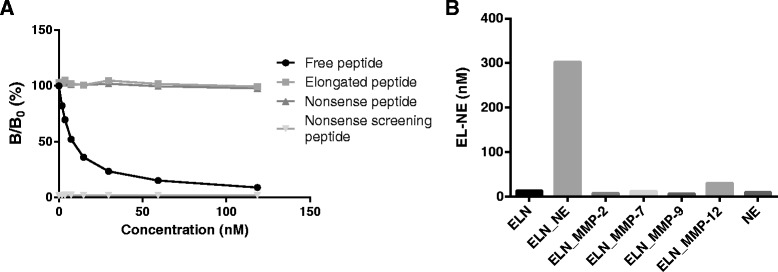
Characterization and Specificity of the EL-NE Monoclonal Antibody. **(A)**: Competitive ELISA showing inhibition of free peptide (GGPGFGPGVV), elongated peptide (GGPGFGPGVVG), nonsense peptide (VGAGVPGLGV) and nonsense screening peptide (Biotin-KK- VGAGVPGLGV). **(B)**: EL-NE fragment levels after 48 hours *in vitro* incubation of intact human elastin separately in buffer (ELN), elastin incubated in the presence of NE, MMP-2, MMP-7, MMP-9 and MMP-12. NE incubated for 48 hours separately in buffer was added as control. Abbreviations: ELN, elastin; NE, Neutrophil elastase; MMP, matrix metalloproteinase.

The inhibition of the EL-NE monoclonal antibody in a competitive ELISA assay with the free-, elongated- and nonsense peptide and nonsense screening peptide is presented in Figure 1A. The EL-NE monoclonal antibody could be inhibited by the free peptide by 91% but was not significantly inhibited by the elongated- (3%) and nonsense peptide (5%). There was no reactivity between the nonsense screening peptide and the EL-NE antibody.

**Figure 2:**
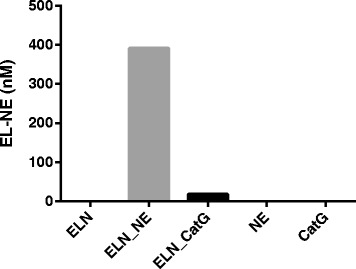
Cross-Reactivity to Cathepsin. EL-NE fragment levels after 24 hours *in vitro* incubation of intact human elastin separately in buffer (ELN) and elastin incubated with CatG and NE. EL-NE levels in NE and CatG separately in buffer are also shown. Abbreviations: ELN, elastin; NE, Neutrophil elastase; CatG, Cathepsin G.

Levels of NE-degraded elastin were assessed in the presence of human elastin cleaved *in vitro* by MMP-2, −7, −9, −12, NE (48 hours) and non-cleaved elastin as well as NE in NE buffer without elastin (Figure 1B). Levels of NE-degraded elastin fragments of elastin incubated in the presence of NE were 2239%, 3168%, 4011%, 2576%, 4709% and 908% higher than elastin incubated without proteases, NE alone, elastin incubated in the presence of MMP-2, MMP-7, MMP-9 and MMP-12 respectively. Levels of elastin *in vitro* cleaved with NE were also compared with Cat-G cleavage of elastin and intact Cat-G (Figure 2). NE-degraded levels of elastin in the presence of the Cat-G, NE enzymes alone and intact elastin after 24 hours incubation were below the lower limit of detection of the assay. Levels of NE-degraded elastin in elastin incubated with NE were 2002% higher than elastin incubated with Cat-G.

Following the selection of EL-NE as a suitable assay, a range of validation tests were performed. The technical validation data for EL-NE is listed in Table 1. EL-NE has a lower limit of detection and quantification of 0.90 nM and 1.9 nM respectively. For human serum, heparin and citrate plasma the EL-NE neoepitope had an analyte recovery percentage of 85% to 104%. The analyte recovery results included samples diluted in assay buffer, samples subjected to four freeze/thaw cycles and samples stored at 4°C for 24 hours. Recovery after 24 hours storage at 20°C was 77% and is not regarded as acceptable, as the limit is 80%. The variation in levels of NE-generated elastin fragments in eight different samples during 10 separate runs was 11% with a 7% intra-assay variation per run.

**Table 1:** **Technical validation of the EL-NE assay**

**Technical validation step**	**EL-NE**
Detection range	0.90 nM – 119 nM
Lower limit of quantification	1.9 nM
Intra-assay variation	7%
Inter-assay variation	11%
Dilution range of serum samples	1:2 (recommended)
Dilution range of plasma samples	1:2 (recommended)
Dilution recovery in serum	104%
Dilution recovery in plasma (Heparin)	86%
Dilution recovery in plasma (Citrate)	108%
Freeze-thaw recovery in serum	96%
Freeze-thaw recovery in plasma (Heparin)	87%
Freeze-thaw recovery in plasma (Citrate)	104%
Analyte stability serum (24 h, 4°C/20°C)	88%/77%
Analyte stability plasma (Heparin) (24 h, 4°C/20°C)	95%/111%
Analyte stability plasma (Citrate) (24 h, 4°C/20°C)	85%/77%

### NE elastin activity is increased during excessive lung remodeling

**Figure 3:**
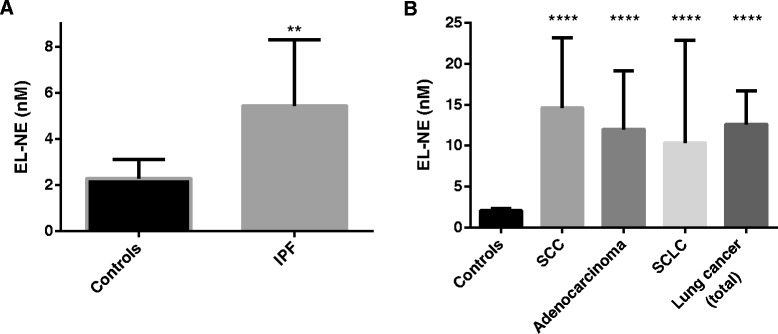
Degradation of Elastin in IPF and lung cancer. **(A)**: EL-NE fragment levels in serum from patients with IPF (n = 10) compared with controls (n = 9). ** p < 0.01. **(B)**: EL-NE fragment levels in serum from patients diagnosed with lung cancer (n = 40 in total), SCC (n = 16), adenocarcinoma (n = 16) and SCLC (n = 8) compared with controls (n = 12). **** p < 0.0001. Groups were compared by T-test with Welch correction. Data are shown as the geometric mean (95% CI). Abbreviations: IPF, idiopathic pulmonary fibrosis; SCC, squamous cell carcinoma; SCLC, small cell lung carcinoma.

Mean serum levels of NE-degraded elastin were 137% higher in IPF patients (5.4 nM) than in controls (2.3 nM, p =0.002, Figure 3A). Mean levels of NE-generated elastin were 510% higher in serum from patients diagnosed with lung cancer (12.6 nM) than in controls (2.1 nM p < 0.0001, Figure 3B). Analysis by cancer subtypes showed NE-degraded elastin levels were 607% higher in squamous cell carcinoma (14.6 nM), 481% higher in adenocarcinoma (12.0 nM) and 402% higher in small cell lung cancer (10.3 nM) than in healthy matched controls.

The EL-NE-B assay did not show elevated NE-generated levels of elastin in IPF (see Additional file 2, Appendix 2: Figure S2A) although it demonstrated high specificity towards its free peptide and elastin cleaved with NE. The EL-NE-B program however was thereafter terminated.

## Discussion

To our knowledge this is the first quantification of an NE-generated elastin fragment in human serum. The main findings of this study were: 1) the selection and quantification of a unique NE-generated elastin fragment; 2) development of the EL-NE antibody which was specific towards a NE cleavage site on elastin and not towards other proteases; 3) development of a competitive ELISA assay for the assessment of NE-degraded elastin in serum; 4) demonstration that levels of NE-degraded elastin were significantly elevated in serum from IPF and lung cancer patients compared with healthy controls.

### Selection of the EL-NE assay

The antibody for the EL-NE assay was selected from a total of 60 antibodies. Besides that used in the EL-NE assay, other promising antibodies were developed towards NE-specific degraded elastin. As an example EL-NE-B (see Additional file 2) demonstrated high specificity towards its free peptide and elastin cleaved with NE. However the clinical relevance of EL-NE-B was low as it could not discriminate between IPF and healthy controls. Furthermore, using the EL-NE-B assay, NE-degraded elastin was only detectable in very low levels in serum from healthy and diseased patients.

### Technical validation and specificity of EL-NE

The low inhibition by the elongated peptide demonstrated that the antibody only recognizes the neoepitope occurring after proteolytic cleavage between Val334 and Gly335. The high levels of the EL-NE fragment in elastin cleaved *in vitro* with NE, compared with MMPs or Cat-G, demonstrated that the EL-NE assay is specific for NE-degraded elastin and that the EL-NE fragment is not a total protein marker since reactivity with intact elastin was minimal. The selected NE cleavage site on elastin is not adjacent to common proteases such as trypsin or chymotrypsin. The levels of the elastin fragment could be recovered after four freeze/thaw cycles and 24 hours storage and the NE-degraded elastin levels decreased in proportion to the number of times samples were diluted. Thus we developed an assay that quantifies a specific NE-derived elastin fragment.

When quantifying the effectiveness of NE inhibitors in pulmonary studies the determination of NE activity often includes the isolation of neutrophils, mainly from the blood [17]. After addition of substrates, such as methoxysuccinyl-Ala-Ala-Pro-Val-p-nitroanilide the NE activity can be quantified. The development and production of NE substrates are considerably faster than monoclonal antibodies. However, the EL-NE assay does not depend on cell extraction and can be applied directly to serum samples. Cell and protease extraction is a significant source of variation and the process may affect NE activity. As demonstrated in this study, the EL-NE assay may recover the majority of analytes after a series of freeze/thaw cycles and storage above freezing temperatures. It is questionable whether ECM proteases stored in serum could maintain the majority of their activity during freeze/thaw cycles.

### Neutrophil elastase activity in IPF

The increased levels of NE-degraded elastin in IPF patient samples indicated that NE-specific degradation of elastin may have a relevant diagnostic role within IPF. In IPF the role of MMPs has received much focus [5,18] but NE activity in IPF can also be an indicator of survival rate [5]. The EL-NE IPF data corresponds well with the discovery that IPF patients with a low BALF neutrophil cell percentage were more likely to survive than IPF patients with a higher percentage [5,19]. The increase in neutrophils in IPF patients may explain the higher levels of circulating elastin fragments in IPF patients compared with controls. EL-NE fragment levels of 2 nM in the controls may be caused by background ECM remodeling combined with matrix effects from the serum. Further studies with control and patient samples obtained with identical standard operating procedures are needed to investigate elastin fragment levels in healthy controls. As the formation of elastin mostly occurs during the younger years increased levels of NE-degraded elastin fragments may be an indicator of imbalanced tissue remodeling, a significant contributor to IPF, in adults. Larger longitudinal studies may reveal the prognostic potential for the EL-NE assay in IPF.

### NE activity in lung cancer

Elevated levels of NE have been coupled with cancer invasion and metastasis in lung cancer [6,11] and most likely explains the increased levels of NE-generated elastin fragments in lung cancer patients. High NE proteolytic activity on elastin may therefore be related to the excessive ECM turnover observed during the progression of lung cancer.

### Limitations

All clinical samples were collected in cross-sectional studies. Longitudinal studies, with several time points and a larger representation of varying disease severity would enable a better description of NE-degraded elastin as a marker of excessive ECM lung turnover. Controls were not matched according to race or smoking history and were collected at different centers than the disease samples. The clinical validation of EL-NE was performed in serum. Future studies should include BALF and plasma samples.

## Conclusions

In conclusion we have utilized specific NE-elastin cleavage sites to develop a non-invasive ELISA assay (EL-NE) to assess levels of NE-generated elastin fragments. The EL-NE analyte can be recovered after dilution, freeze/thaw cycles and temporary storage above freezing temperature. The developed EL-NE assay was specific towards elastin cleaved *in vitro* with NE. We conclude that the quantification of the EL-NE analyte may be a marker of the increased tissue remodeling observed in pulmonary disorders such as IPF and lung cancer.

## Additional files

Additional file 1:
**Appendix 1, Table S1:** Patient Demographics and Clinical Profiles of Cancer and IPF Patients. Additional file 2:
**Appendices 1-2, Figure S1 and Figure S2.** Appendix 1: EL-NE-B Methods; Appendix 2: EL-NE-B Data; Figure S1: Characterization and specificity of the EL-NE-B monoclonal antibody; Figure S2: The EL-NE-B Assay in IPF.

## Abbreviations

BALF: Bronchoalveolar lavage fluid
CatG: Cathepsin G
CI: Confidence interval
COPD: Chronic obstructive pulmonary disease
CV: Coefficient of variation
ECM: Extracellular matrix
HRP: Horseradish peroxidase
IPF: Idiopathic pulmonary fibrosis
MMP: Matrix metalloproteinase
NE: Neutrophil elastase
TMB: Tetramethylbenzinidine
