# Supplementary material for: Serological assessment of neutrophil elastase activity on elastin during lung ECM remodeling
Source: BMC Pulm Med. 2015 May 3;15:53. doi: 10.1186/s12890-015-0048-5 (PMC4426538; doi:10.1186/s12890-015-0048-5)
Supplement: Additional file 1: — Appendix 1, Table S1: Patient Demographics and Clinical Profiles of Cancer and IPF Patients. [file 12890_2015_48_MOESM1_ESM.pdf]

Additional File 2

Serological Assessment of Neutrophil Elastase Activity during Lung ECM Remodeling

[£12]Jacob H. Kristensen, [1]Morten A. Karsdal, [1]Jannie M.B. Sand, [1]Nicholas Willumsen, Claudia Diefenbach[3], Birte Svensson, Per Hägglund, [1]Diana J. Oersnes-Leeming

[£]Corresponding author: Jacob H. Kristensen jhk@nordicbioscience.com

[1]Nordic Bioscience A/S, Herlev, Denmark

[2]The Technical University of Denmark, Department of Systems Biology, Denmark

[3]Boehringer-Ingelheim Pharma GmbH, Biberach, Germany

Contents

Appendix 1:

Table S1: Patient Demographics and Clinical Profiles of Cancer and IPF Patients

Appendix 1

.

Group	n	Gender, % females	Tumor stage (n)				Smoking status			Age, mean ± SD (range)
			I	II	III	IV	never	ever	unknown	
Lung cancer, all	40	25%	13	12	12	3	3	35	2	59±10 (46-82)
SCLC	8	25%	2	1	4	1	-	7	1	61±12 (46-82)
NSCLC	32	25%	11	11	8	2	3	28	1	60±9 (46-80)
Adenocarcinoma	*16*	*37.50%*	*4*	*6*	*6*	-	-	*15*	*1*	*57±10 (46-80)*
Squamous cell carcinoma	*16*	*12.50%*	*7*	*5*	*2*	*2*	*3*	*13*	-	*63±6 (53-73)*
IPF (FEV 63-68 %)	10	20%	-	-	-	-	9	1	-	74±5 (67-83)

Table S1: Patient Demographics and Clinical Profiles of Cancer and IPF Patients. Abbreviations: FEV, forced expiratory volume; IPF, idiopathic pulmonary fibrosis; NSCLC, non-small-cell lung carcinoma; SCLC, small cell lung carcinoma.
