# Supplementary material for: Serological assessment of neutrophil elastase activity on elastin during lung ECM remodeling
Source: BMC Pulm Med. 2015 May 3;15:53. doi: 10.1186/s12890-015-0048-5 (PMC4426538; doi:10.1186/s12890-015-0048-5)

Additional File 1

Serological Assessment of Neutrophil Elastase Activity during Lung ECM Remodeling

[£12]Jacob H. Kristensen, [1]Morten A. Karsdal, [1]Jannie M.B. Sand, [1]Nicholas Willumsen, Claudia Diefenbach[3], Birte Svensson, Per Hägglund, [1]Diana J. Oersnes-Leeming

[£]Corresponding author: Jacob H. Kristensen jhk@nordicbioscience.com

[1]Nordic Bioscience A/S, Herlev, Denmark

[2]The Technical University of Denmark, Department of Systems Biology, Denmark

[3]Boehringer-Ingelheim Pharma GmbH, Biberach, Germany

Contents

Appendix 1: EL-NE-B Methods

Appendix 2 EL-NE-B Data

Figure S1: Characterization and specificity of the EL-NE-B monoclonal antibody

Figure S2: The EL-NE-B Assay in IPF

Appendix 1

EL-NE-B Methods

Immunization procedure

The immunization procedure for EL-NE-B followed the same procedures as EL-NE with the exception of the Freund's incomplete adjuvant (KLH-CGG-VGAGVPGLGV) which was acquired from Chinese Peptide Company (Beijing, China)

Characterization of clones

The characterization of the EL-NE-B clones was performed similarly to the characterization of the EL-NE clones with the exception of the biotinylated peptide (Biotin-KK- VGAGVPGLGV), the free peptide (VGAGVPGLGV) and the elongated peptide (VGAGVPGLGVG). All used peptides were produced by Chinese Peptide Company (Beijing, China).

EL-NE-B Assay protocol

The EL-NE-B assay protocol followed the EL-NE protocol with the exception of the above-mentioned peptides.

Clinical validation of EL-NE-B

Levels of NE-degraded elastin were determined with the EL-NE-B assay in serum from IPF patients. The diagnosed samples and controls were identical to those investigated with the EL-NE assay in the main manuscript. Additional patient demographics and clinical information is presented in table S1 (See Additional file 2, Appendix 1).

Statistical analyses

The geometric means (95% CI) of the NE-degraded elastin fragments quantified by the EL-NE-B assay, in diagnosed patients, were compared with their respective controls using the two-sided non-parametric Mann Whitney test. All statistical analysis was performed in MedCalc from MedCalc Software (Ostend, Belgium). Results were considered statistically significant if $p < 0.05$.

Appendix 2

EL-NE-B Data

Reactivity and specificity of EL-NE-B

The inhibition of EL-NE-B, in a competitive ELISA assay, with the free and elongated peptide is presented in Figure S1A. The EL-NE-B antibody was inhibited by the free peptide by 77% but not by the elongated peptide (0%).

EL-NE-B levels were assessed in the *in vitro* cleavage of human elastin with MMP-2, -7,-9, NE and non-cleaved elastin as well as NE alone (Figure S1B). EL-NE-B levels in elastin cleaved with NE were over 1700% higher than EL-NE-B levels in the remaining material.

Clinical relevance of EL-NE-B

There was no significant difference in EL-NE-B levels in serum from IPF patients and healthy controls (Figure S2).

Figure S1: Characterization and specificity of the EL-NE-B monoclonal antibody. (A): Competitive ELISA showing inhibition of free peptide (VGAGVPGLGV) and elongated peptide (VGAGVPGLGVG). (B): EL-NE-B levels after in vitro cleavage of intact human elastin and elastin cleaved with MMP-2, MMP-7, MMP-9 and NE. The EL-NE-B level in NE separately is also shown. Abbreviations: ELN, elastin; NE, Neutrophil elastase; MMP, metalloproteinase

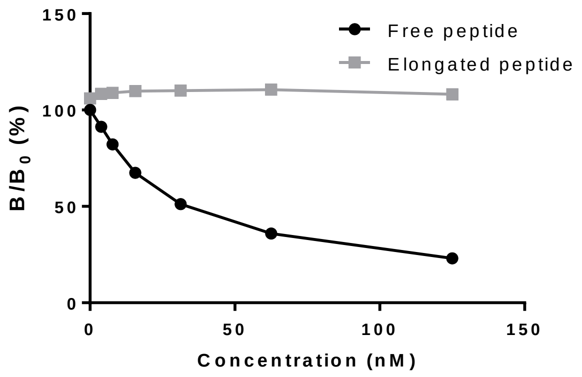

A

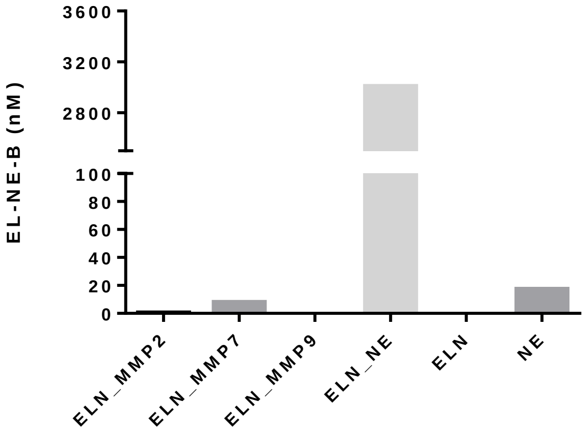

B

Figure S2: The EL-NE-B Assay in IPF. EL-NE-B levels in serum from patients with IPF (n=10) compared to controls (n=9). Groups were compared by T-test with Welch correction. Data is shown as the geometric mean (95 % CI). Abbreviations: IPF, idiopathic pulmonary fibrosis; ns, non-significant.

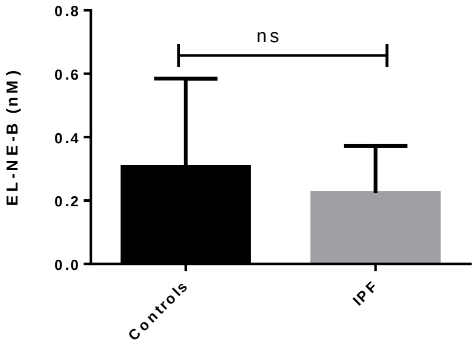

Supplement: Additional file 2: — Appendices 1-2, Figure S1 and Figure S2. Appendix 1: EL-NE-B Methods; Appendix 2: EL-NE-B Data; Figure S1: Characterization and specificity of the EL-NE-B monoclonal antibody; Figure S2: The EL-NE-B Assay in IPF. [file 12890_2015_48_MOESM2_ESM.pdf]
